# Mast Cell Involvement in the Pathogenesis of Selected Musculoskeletal Diseases

**DOI:** 10.3390/life13081690

**Published:** 2023-08-05

**Authors:** Łukasz Gutowski, Szymon Kanikowski, Dorota Formanowicz

**Affiliations:** 1 Department of Medical Chemistry and Laboratory Medicine, Poznan University of Medical Sciences, Rokietnicka 8, 60-806 Poznan, Poland; skanikowski@ump.edu.pl; 2 Department of Stem Cells and Regenerative Medicine, Institute of Natural Fibres and Medicinal Plants—National Research Institute, Kolejowa 2, 62-064 Plewiska, Poland

**Keywords:** mast cell, MC, rheumatoid arthritis, RA, spondyloarthritis, psoriatic arthritis, gout, tendinopathy, hypermobile Ehlers–Danlos syndrome, hEDS

## Abstract

In recent years, there has been a noteworthy revival of interest in the function of mast cells (MCs) in the human body. It is now acknowledged that MCs impact a wide array of processes beyond just allergies, leading to a shift in research direction. Unfortunately, some earlier conclusions were drawn from animal models with flawed designs, particularly centered around the receptor tyrosine kinase (Kit) pathway. Consequently, several subsequent findings may have been unreliable. Thus, what is now required is a re-examination of these earlier findings. Nevertheless, the remaining data are fascinating and hold promise for a better comprehension of numerous diseases and the development of more effective therapies. As the field continues to progress, many intriguing issues warrant further investigation and analysis. For instance, exploring the bidirectional action of MCs in rheumatoid arthritis, understanding the extent of MCs’ impact on symptoms associated with Ehlers–Danlos syndrome, and unraveling the exact role of the myofibroblast–mast cell–neuropeptides axis in the joint capsule during post-traumatic contractures are all captivating areas for exploration. Hence, in this review, we summarize current knowledge regarding the influence of MCs on the pathogenesis of selected musculoskeletal diseases, including rheumatoid arthritis, spondyloarthritis, psoriatic arthritis, gout, muscle and joint injuries, tendinopathy, heterotopic ossification, and Ehlers–Danlos syndrome. We believe that this review will provide in-depth information that can guide and inspire further research in this area.

## 1. Introduction and Methodology

Since their discovery in the second half of the 19th century, MCs have mainly been studied regarding their role in allergies. However, the turn of the century saw a surge of interest in their impact on diseases related to joints, muscles, and tendons. Nowadays, one can encounter both far-fetched opinions about the crucial role of MCs in the majority of disease entities and opinions trying to tone down this enthusiasm, pointing out many inconsistencies, even errors, in many of the conducted studies. 

**Figure 1:**
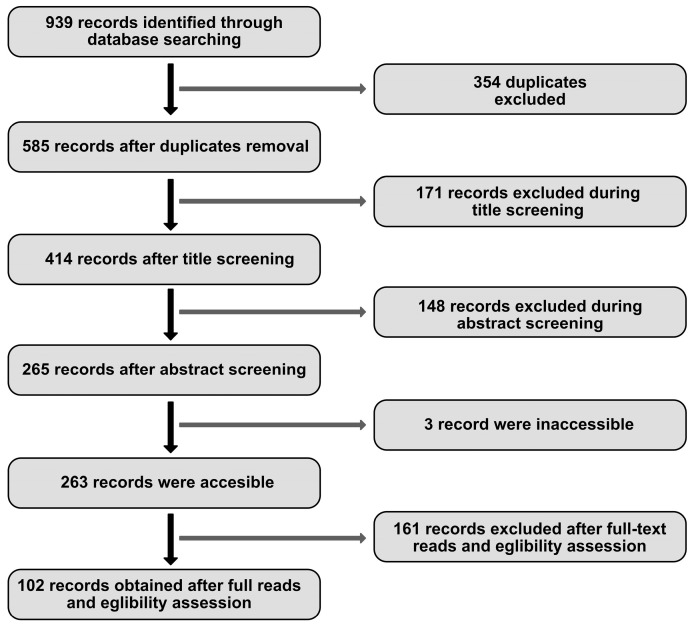
Literature search flow diagram.

This paper aims to present current knowledge on the impact of MCs on the development of selected musculoskeletal diseases and provide, where possible, differing views on these issues. For this purpose, a literature search of publications released since 2010 was carried out on 1 February 2023 using the PubMed database. The main query entered into the search engine was “musculoskeletal diseases AND mast cell”, yielding 477 results. To ensure that the search would include musculoskeletal entities, which were also a point of interest for the authors, the following queries were also entered: “muscular diseases AND mast cell”; “rheumatoid arthritis AND mast cell”; “gout AND mast cell”; “psoriatic arthritis AND mast cell”; “myopathy AND mast cell”; “spondyloarthritis AND mast cell”; “Ehlers-Danlos Syndrome AND mast cell”. A total of 939 records were identified via PubMed search. After removing duplicates, 585 results remained. The next step was to check the results, which was performed independently by two researchers, and discrepancies were discussed. Finally, 483 articles were excluded based on titles (*n* = 171), abstracts (*n* = 148), full-text reads (*n* = 161), and due to being inaccessible (*n* = 3) (see Figure 1). Articles addressing issues unrelated to this review were discarded, which included neoplasms, animal diseases, skin diseases, oral diseases, neurological diseases, dietary recommendations, studies focusing exclusively on an active substance, and studies on which scientific quality was found to be objectionable. Due to the voluminous nature of the work, it was also decided not to describe bone diseases, including osteoarthritis. One hundred two papers were selected and used as the basis of this review.

## 2. Mast Cells Biology

MCs are far more complex than they are commonly given credit for. Understanding the fundamental functions of these cells is essential to grasp their impact on musculoskeletal conditions. While considered part of the innate immune system, MCs are amongst the most important immune cells sensing danger signals; their role is positioned between the innate and acquired immune systems. While their involvement in immunoglobulin E (IgE)-mediated allergic inflammation is well known, they have also been implicated in various non-allergic inflammatory processes, acting as an early warning system for invaders and coordinating and directing the immune response [1,2]. MCs are developed from CD34+ hematopoietic precursor cells, which occur in the bone marrow and circulate in the blood immaturely. They can fully differentiate and mature only after taking residence in specific tissues. They can be activated by factors such as IgG–antigen complexes, pathogen-associated molecular patterns (PAMPs), complements, cell–cell contact, cytokines, certain drugs, hormones, and physical activators such as temperature and pressure. They reside not only in tissues such as the skin, gut, and respiratory and urinary tract that form the barriers between the self and the environment but also within lymph nodes, near blood vessels, and nerves, giving them the perfect location for early detection and safeguarding the organism [3].

MCs secrete a variety of products: proteases (tryptase, chymase, carboxypeptidase, MCP-1, MCP-2), proteoglycans (heparin, chondroitin sulfate), proteins (CRH, osteopontin, thymic stromal lymphopoietin), biogenic amines (histamine, serotonin), growth factors (PDGF, VEGF, FGF2, bFGF, NGF, PAF), and cytokines (TNFα, lymphotactin IL-1β, IL-3, IL-4, IL-5, IL-6, IL-9, IL-10, IL-12 IL-13, IL-16, IL-17A, IL-18, IL-23 IFN-α, IFN-β, IFN-γ). Moreover, MCs possess the ability to synthesize plasma-membrane-derived lipid mediators, including prostaglandin D2 (PGD2) and leukotriene B4 (LTB4), for various biological actions; in addition, they are proficient antigen-presenting cells [1]. The critical element to release these mediators is degranulation. It is a complex process involving membrane fusion and various proteins, depending on whether it is triggered by allergic or non-allergic reactions [4]. In a non-allergic reaction, degranulation is initiated by the fusion of granules with the plasma membrane, but this process depends on the interaction between vesicle-associated membrane protein 8 (VAMP8) and 7 (VAMP7) exclusively present on the granule membrane of the mast cell. In non-allergic reactions, there are three main pathways of degranulation activation:

(a) Mas-related G-protein-coupled-ligand receptor member X2 (MRGPRX2) binding results in Inositol Phosphate-Phospholipase C (PI-PLC) activation, thus leading to the formation of Inositol trisphosphate (IP_3_) and the opening of Ca^2+^ channels, increasing intracellular Ca^2+^ concentration and starting degranulation.
(b) A benzoyl ATP binding activates ion channels, resulting in rapid nonselective ion influx, including Ca^2+^, which opens large plasma membrane pores and causes degranulation [4,5].
(c) Activation by physical contact with activated T-cell membranes and by released microvesicles stimulates extracellular signal-regulated kinase 1/2 (ERK1/2) activation and causes the expression of cytokines, chemokines, adenosine, and growth factors. Adenosine binds to A3 receptors (A3R) and initiates ERK1/2 signaling. It is suspected that neuroblastoma RAS viral oncogene homolog (N-Ras) may be activated by this method, resulting in ERK activation associated with increased cytokine release. Also, this pathway results in tumor necrosis factor-alpha (TNF-α) release, which stimulates the production of the granule-associated enzyme matrix metallopeptidase 9 (MMP-9) [2]. MMP-9 is produced from proMMP-9 with the use of chymase present in mast cells. The stimulation of mast cells by inflammation and oxidative and mechanical stress causes the release of chymase from the granules of mast cells, changing the pH in which chymase is operating from 5,5 in mast cell granules to 7-9, creating ideal conditions for chymase to employ. Moreover, because of endogenous chymase inhibitors (α-antitrypsin and α-antichymotrypsin) found in blood, the enzymatic function of chymase disappears immediately; thus, only activated mast cells can exert its enzymatic function [6].

Additionally, it is pertinent to highlight the interplay between regulatory T cells (T-reg) and mast cells, as their interaction plays a pivotal role in achieving optimal inflammation suppression. This relationship can be described as pseudo-symbiotic, wherein T-reg cells attract mast cells by secreting Interleukin 9 (IL-9), a growth and activation factor for mast cells. In return, mast cells release IL-2, a critical factor for the proliferation and function of T-reg cells. This pathway of mast cell activation was found to be Ig-E-dependent, like in an allergic reaction. A similar connection between T-reg cells, IL-9, and MCs is suspected in immune suppression in B-cell non-Hodgkin’s lymphoma and murine lymphoma. However, the precise mechanism causing such suppression has not been fully discovered yet [2].

**Figure 2:**
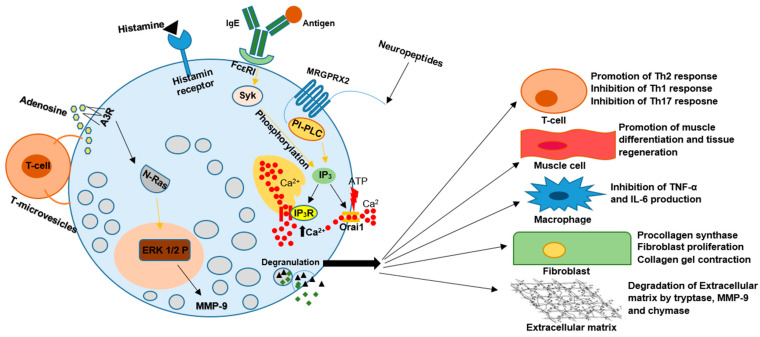
The pleiotropic nature of MCs functions result in their involvement in the pathogenesis of many disease entities. Various factors such as IgG–antigen complexes, PAMPs, complements, physical activators, cell–cell contact, cytokines, certain drugs, neuropeptides, and hormones can activate MCs during degranulation. MCs secrete vasoactive amines, cytokines, and proteases, including tryptase, chymase, and carboxypeptidase. They can also synthesize plasma-membrane-derived lipid mediators, including PGD2 and LTB4. All of the above can change the activity of other immune cells, muscle cells, fibroblasts, and many more. The induced changes have the potential to result in an aggravation or inhibition of the development of many disorders.

In an allergic reaction, mast cells are activated by recognition of an Ag-specific IgE bound to the α subunit of FcεRI expressed on the cell surface. Protein tyrosine kinase Lyn provides a recognition signal allowing for intracellular signal transformation by transphosphorylation of the Fcγ receptor I β (FcεRI β) and γ subunits. Due to alternative splicing, two isoforms of Lyn kinase exist in MCs—Lyn A (56 kDa) and Lyn B (53 kDa). Tyrosine kinases are crucial for MCs in allergic reactions by coimmunoprecipitation with FcεRI, thus leading to the phosphorylation of crucial substances such as Syk tyrosine kinase. This fact allows phospholipase C (PLC)γ to catalyze the hydrolysis of IP_3_ and, in effect, trigger calcium influx from the extracellular environment via Orai1/CRACM calcium channels. Calcium influx and PLC are essential to release allergic mediators stored in granules, such as histamine and the de novo synthesis of cytokines and eicosanoids by MCs. Lyn B is less effective in triggering calcium responses and MC degranulation but is equivalent to Lyn A in total cellular tyrosine phosphorylation and FcεRI phosphorylation. Lyn A shows more robust calcium responses and degranulation than Lyn B and is better at promoting the interaction of PLCγ with phospho-LAT (see Figure 2) [4,5].

The information presented above provides an overview of the fundamental functions of MCs as cells vital in inflammatory and other immune responses. Apart from their well-established association with allergies and increased vascular permeability, MCs also play a role in responding to bacterial and viral infections, parasite infestations, and systemic diseases. Additionally, MCs play a crucial part in tissue remodeling, angiogenesis, and wound healing. There are compelling indications that mast cells are implicated in the pathogenesis of numerous musculoskeletal diseases, such as rheumatoid arthritis, psoriatic arthritis, scleroderma, multiple sclerosis, and many others [1,4]. In inflamed tissues, MCs utilize the aforementioned mediators to modulate the actions of cells like neutrophils or T-cells and present antigens to the latter. MCs can interact with different types of T-cells, thus showing not only proinflammatory but also anti-inflammatory effects. Interestingly, there is even suspected to be a regulatory mast cell population [2]. These dual capabilities of MCs, though not fully understood or well documented, could potentially explain why many high-quality studies report conflicting results regarding the impact of MCs on specific diseases.

## 3. Rheumatoid Arthritis

Rheumatoid Arthritis (RA) is a chronic autoimmune disease manifested by joint swelling and tenderness, which can progress to disability, impacting both the physical and mental well-being of the patient. It affects 5 to 10 per 1000 people and disproportionally affects the female population [7,8]. Recently, increasing attention has been focused on uncovering the involvement of mast cells in the pathophysiology of this disease. Nevertheless, certain studies conducted on mice have raised some valid concerns.

### 3.1. Mouse Models

To gain a comprehensive understanding of the challenges associated with animal research on the impact of MCs in RA pathogenesis, it is essential to familiarize oneself with the mouse models commonly employed in many studies and be aware of their limitations. To study RA pathogenesis, MC-deficient mouse strains were generated. The initial MC-deficient mouse type was based on a mutation in the gene encoding receptor tyrosine kinase (Kit). The Kit pathway, initiated by Kit and its ligand SCF, is essential for MC maturation, survival, and activation. Standard models based on this mutation are Kit^W/Wv^, Kit^W-sh/W-sh^, and WCB6F1-Mgf^Sl/Sl-d^—all of them result in MC deficiency. Unfortunately, Kit has pleiotropic functions, such as regulating metabolic responses and pain. It also affects the development of hematopoietic stem and progenitor cells, red blood cells, neutrophils, intestinal pacemaker cells, melanocytes, and germ cells. Kit^W/Wv^ mice suffer from anemia, neutropenia, and show impairment in lymphocyte development. WCB6F1-Mgf^Sl/Sl-d^ mice manifest similar abnormalities to the WBB6F1-Kit^W/Wv^ mice. Kit^W-sh/W-sh^ are fertile and have milder abnormalities, namely splenomegaly, neutrophilia, and thrombocytosis [9]. Kit-independent MC-deficient mice provided researchers with a valuable tool to investigate the precise role of mast cells in immunological and inflammatory reactions. However, it has been proven that the selective absence of mast cells has different consequences than the combined mast cells and Kit deficiency. As Kit exerts a widespread influence on various organism functions, it became challenging to draw definitive conclusions solely on the impact of mast cells on experiments. To distinguish Kit deficiency from MC deficiency, mast-cell-reconstitution Kit mutants have been reconstituted with bone marrow-derived cultured mast cells (BMMCs) [10]. Because of the abovementioned reasons, considerable efforts have been dedicated to generating mouse strains that are exclusively deficient in mast cells without affecting other immune system components.

The Cre-mediated MC eradication mouse line (Cre-Master, Cpa3^Cre/+^) was generated by targeted insertion of Cre-recombinase into the mast cell Cpa3 gene. High expression of the Cre recombinase exerts genotoxicity, leading to MC eradication. Except for the reduction in basophil level, mice still have a proper immune system but cannot initiate IgE-mediated allergic responses. Nevertheless, they are fully vulnerable to antibody-induced autoimmune arthritis. Similarly, mouse Cpa3^Cre^; Mcl-1^fl/fl^ strain (Cpa3-Cre) has a strongly reduced number of MCs (92% to 100%), a milder decrease in the number of basophils (58% to 78%), but impaired IgE-mediated responses [9,10].

To develop a strain with no abnormalities in the cellular composition of the spleen, blood, skin, and bone marrow, the Mcpt5^Cre^ mouse line was mated with the R-DTA strain. Mcpt5Cre R-DTA mice resulted in the MC-specific expression of DTA and, thus, in a toxin-mediated constitutive reduction of 90% of MCs in the peritoneum and skin [9].

An opposite approach was used to generate mice lacking A20—specifically, in connective-tissue-type MCs (Mcpt5Cre A20^F/F^). The MC-specific ablation of A20, a negative feedback regulator, exaggerates inflammatory MC signaling but does not provoke anaphylactic reactions. As it turns out, hyperactive A20-deficient MCs cause an earlier onset and worsening of Collagen-Induced Arthritis (CIA) symptoms [11].

To date, numerous methods for inducing arthritis to simulate RA have been developed and investigated in the aforementioned mouse models. One of them is the transfer of serum from K/BxN transgenic mice (serum-transfer-induced arthritis, STIA), which produce autoantibodies against the glucose-6-phosphate isomerase (GP6I), inducing an arthritic phenotype. Kit^W/Wv^ and Mgf^Sl/Sl-d^ mice are protected from arthritis induced in such a way, but reconstituting Kit^W/Wv^ mice with BMMC brought back the susceptibility to the disease. It was also reported that neutrophil transfer could be sufficient to restore K/BxN-passive arthritis in Kit^W/Wv^ mice. Some of the disagreements could be a consequence of how many arthritogenic antibodies were given to each animal and on what day the arthritis was evaluated. Despite that, Kit^W-sh/W-sh^ mice were not protected from STIA, probably because of impaired neutrophil levels. Similar conclusions were drawn by Pitman et al.; in their research, collagen-induced arthritis (CIA), which is induced by immunizing with type II collagen, was used in wild-type and MC-deficient Kit^W-sh/W-sh^ mice on a C57BL/6 background. The absence of mast cells did not substantially change the disease course. Therefore, immune activation against type II collagen can drive mast-cell-independent pathways, leading to synovitis in Kit^W-sh/W-sh^ mice [12,13,14,15].

Schubert et al. identified problems within the studies carried out on Cre-mediated mice. According to their research, MC-competent Cre^−^; iDTR^+^ mice and MC-depleted cpt5-Cre^+^; iDTR^+^ mice, which received K/BxN sera, showed equal levels of arthritis. However, in STIA, arthritis is induced in a T-cell-independent manner, while in humans, RA can be activated in both humoral and cellular ways. Nonetheless, the same mice after the induction of CIA proved MCs to be critically relevant, not only because the absence of MCs resulted in lower inflammation but also in the loss of T cell expansion and reduced T cell cytokine levels [16]. However, Cunin et al. stated that the mechanisms behind mouse models of MC influence are much more nuanced, and the role of MCs in a particular setting can vary depending on the need for amplifying the underlying inflammatory trigger. It is also possible to learn from the same work about another critical factor—megakaryocytes, which, through IL-1 secretion, can activate synovial fibroblasts. The presence of these factors restores the susceptibility of Kit^W/Wv^ mice to RA induction [17].

### 3.2. MCs Types in RA

MCs are resident cells in the healthy synovial joint, but in the synovium of RA patients, MC hyperplasia can be observed [18], although this feature is common in many autoimmune diseases [19]. Recent studies examining the synovium in early RA have revealed distinct synovitis phenotypes based on MC infiltration density [18]. Patients with high MC presence have substantially increased CRP levels, high lymphocyte counts in the RA synovium, and high disease activity scores. However, data concerning the correlation of ESR with the amount of MC are contradictory and study-dependent [18,20,21]. Additionally, two genes regarded as RA’s diagnostic markers—LSP1 and GNLY—are also associated with MCs, underscoring the relevance of these cells in RA pathogenesis [8].

Nevertheless, the detailed impact on the RA mechanism has not been fully elucidated; even their role in the different phases of RA development remains a topic of debate [22]. Two main types of mast cells can be distinguished: the tryptase-chymase double-positive cell (MC_TC_) and tryptase-only positive cell (MC_T_). MC_T_ is associated with inflammation, especially in the early stages of RA, while MC_T_ expansion followed by MC_TC_s is associated with tissue remodeling, characteristic of established or chronic disease. MC_TC_s are also the primary subtype in the normal synovium [19]. It noteworthy that RA patients in ultrasound-defined remission had significantly reduced synovial mast cell density compared to patients with clinically active RA [23].

### 3.3. Autoantibodies and Receptors

In rheumatoid arthritis, anti-citrullinated protein antibodies (ACPA) are an essential group of autoantibodies. They recognize various peptides in which arginine has been modified into citrulline by the family of Peptidyl Arginine Deiminase (PAD) enzymes, which can be produced in RA by macrophages, dendritic cells, neutrophils, and mast cells [24].

ACPA can bind to antigens and activate the complement system, activating mast cells through the cleavage product C5a. It is unclear if this pathway leads to autoantibody-mediated mast cell activation in humans. Mast cells can also be activated directly by Fc receptors, particularly Fcγ receptors. Because ACPAs are mostly IgM and IgG isotypes, IgG-ACPA binding to receptors is thought to play a significant role in autoantibody-induced pathogenesis [19]. IL-33 also enhances this activation by inducing MCs to release IL-10, histamine, and other cytokines associated with type 2 immune responses, such as IL-5 and IL-13 [25].

Mice synovial MCs express the activating FcγRIIIa, which is involved in arthritis induced by the anti-collagen autoantibodies model [19]. In contrast, the Fc receptor β chain, also an FcγRIII component, negatively regulates arthritic inflammation in a mice knock-out model [26]. Nevertheless, human studies showed that synovial MCs of all studied patients could be activated by the cross-linking of ACPA with another receptor—FcγRIIA—which suggests that this is a significant factor in autoantibody-mediated mast cell activation [19]. It agrees with the study by Lee et al., according to which FcRI and FcRII are responsible for the induction of aggregated IgG-dependent activation of synovial MCs obtained from patients with RA [27] and with the study conducted by Suurmond et al., where both human-cultured and synovial mast cells expressed FcγRIIA. However, in the latter, FcγRI was not expressed by synovial mast cells derived from most patients [28].

Nonetheless, it should be taken into consideration that Tsuboi et al. reported that in human-FcγRIIa-expressing, γ-chain-deficient (hFcγRIIa^+^/γ^−^/^−^) mice, stimulation of neutrophils via FcRIIa is not associated with mast cell degranulation and, indeed, proceeds in the absence of these cells [27]. Also, according to Mancardi et al., at least IgG1 and IgG2 antibody isotypes, and two receptors, FcgRIIIA and FcgRIV, contribute to K/BxN-induced arthritis, and at least two cell types other than mast cells are required for this process—monocytes or macrophages, and neutrophils [15].

The molecular processes taking place between FCγ activation and MC degranulation are insufficiently studied. Guma et al. demonstrated using K/BxN C-Jun N-terminal kinase (JNK) 1−/− and Jnk2−/− mice) that JNK1 but not JNK2 is an essential component of FcγR-induced release of proinflammatory mediators, such as IL-1β [29].

Other receptors involved in RA are Toll-like receptors (TLRs). Mast cells express a variety of TLRs, which induce MC activation after being triggered. MCs also express TLR-mediated responses to endogenous ligands released during inflammation, such as TLR-2, TLR-4, and endosomal TLRs [19].

TLR4-mediated signals induce the production of IL-12 by macrophages, mast cells, and Gr-1+ cells, which regulate IL-1b and IFN-g production, suppressing TGF-β production. This regulation of the cytokine network plays a crucial role in joint inflammation [30].

The combination of TLR- and Fcγ-mediated activation enhances cytokine production by human mast cells. Such synergy can dictate the type and extent of immune cells attracted to the inflammation site. The synergy between TLRs or cytokines and the Fc receptor is highly beneficial when pathogen elimination is required but can also lead to the further release of modified autoantigens and TLR ligands, contributing to the chronicity of rheumatoid arthritis [19,28].

### 3.4. Cytokines

Cytokines can not only affect mast cells’ survival but can also activate them. Cytokines such as IL-3, IL-4, IL-5, and IL-33, which are increased in synovial fluid of RA patients, can influence MC proliferation and activity. The stimulation of MCs with cytokines alone contributes more to their proliferation than activation, but the activation effect combined with other factors is powerful. Recently, great attention has been paid to IL-33, which has been shown to have multiple implications on MC functionality. IL-33 is derived mainly from synovial fibroblasts and can regulate the maturation and activation of MC on its own, increasing the number of tryptase-positive and tryptase-chymase double-positive cells [31,32,33]. Via its receptor ST2, lL-33 induces the expression of the activating receptor for the Fc fragment of IgG (FcgRIIa) in human mast cells [25]. It is worth noticing that in vivo, IL-33 exacerbated CIA in ST2^−/−^ mice engrafted with WT but not ST2^−/−^ mast cells [34]. Furthermore, IL-33 also induces osteoclast differentiation from synovial fluid monocytes independent of RANKL but reduces their differentiation in RANKL-induced osteoclastogenesis. IL-33-stimulated MCs induce more osteoclast differentiation, probably by cell–cell contact [32]. Nonetheless, some studies indicate that IL-33 mRNA levels were inversely correlated with proinflammatory markers, such as CD14, CD16 (FcgRIIIA), and TNF. These observations speculate that mast cells, particularly when triggered by IL-33, could dampen the activation of monocytes and modulate the immune response in the synovial tissue of patients with RA [25,35].

Another essential cytokine is IL17A (also called IL-17), which is elevated in the synovial fluid of RA patients. It has been considered lately as one of the potential new targets for treating this disease. IL-17 can induce the production of proinflammatory factors such as IL-6, IL-1, TNF, and matrix metalloproteinases. While, in the peripheral blood of patients with immune-mediated disease, Th17 cells are the most critical source of IL-17, in the joints of RA patients, mast cells have been considered essential producers of this interleukin [36]. This thesis was also confirmed by Hueber et al., who proved that MCs are one of the primary sources of IL17A in established synovitis [37], but in vitro studies did not show such a relationship [38].

### 3.5. MC Granules’ Content

MCs secrete many products, but some of them—like chymase, histamine, and tryptase—are elevated in both the synovial fluid and peripheral blood of RA patients. Since they are mast-cell-specific, it likely reflects local mast cell activation, which is supported by the fact that serum tryptase is correlated with RA disease activity [19,32]. It is believed that many serine proteinases are thought to be involved in rheumatoid arthritis as they are elevated in the synovial fluid of such patients. Proteinases transmit the signal through the Protease-Activated Receptors (PAR) receptors family, allowing them to function as inflammatory mediators and degradative enzymes. Depending on the PAR family member, PAR activation can modulate inflammation and pain. As it turns out, in rats, PAR4 is expressed on synovial MCs, which indicates that they are key players in mediating the signaling effects of serine proteinases. In this case, PAR4 activation resulted in a pronociceptive effect depending on mast cell activation [39].

Tryptase is one of the serine proteinases, characteristic for MC, and it is the main substance stored in MC_T_ (MCs containing only tryptase). Tryptase, along with histamine, is co-responsible for increased vascular permeability during inflammation, and fibroblast and epithelial cell proliferation. It is also one of the compounds involved in MC–neuron communication. As it turns out, injection of this protease into the mice joints results in inflammation and swelling; corresponding studies have proven that mice lacking monocyte chemotactic protein 6, a human tryptase analog, show resistance to antibody-mediated arthritis. That is why tryptase and its receptor, PAR-2, may play a significant role in RA pathogenesis. 

This information sheds light on two processes co-occurring in the tissues of RA patients: the proliferation of RA synovial fibroblasts (RASFs), which contributes to the pathogenesis of RA, and increased production of Fas, which should lead to apoptosis of RASFs. Although in vitro Fas-mediated apoptosis is significant, in vivo, this mechanism of apoptosis induction is limited. This seemingly contradictory information was reconciled when it turned out that RASFs express the receptor for mast cell tryptase (PAR-2) and, thus, are protected from Fas-mediated apoptosis by tryptase in a Rho-kinase-dependent manner. PAR-2 is also expressed in other cells; therefore, it should be investigated if MCs affect them through a tryptase/PAR-2-dependent mechanism in RA patients [4,40].

In addition to tryptase, MCs secrete other serine proteinases, such as chymase and carboxypeptidase A3. Chymase can degrade the extracellular matrix, activate procollagenases, aggravate leukocyte migration, and affect tissue remodeling in diseases like asthma and chronic obstructive pulmonary disease [4]. MC-chymase does not significantly affect normal synovial fibroblast (SFB) growth but induces the proliferation of fibroblast-derived from rats with CIA. CIA-SFB treated with chymase exhibits increased MMP-9 and lowered MMP-2 expression. Because of altered MMP-9 levels, chymase promotes CIA-SFB adherent abilities and invasive migration [41]. Similar conclusions regarding MMP-9 can be drawn based on mouse models in which mMCP-5, an enzyme similar to human chymase, also affected levels of this metalloproteinase [42]. Carboxypeptidase A3 (CPA3), also known as human MC carboxypeptidase A, is a protease highly expressed in MCs. CPA3 is stored in their granules by ionically bounding to heparin-containing serglycin proteoglycans. Besides its primary proteolytic function, CPA3, like the other mediators in MC granules, also affects inflammation, pain, vasodilation, angiogenesis, coagulation, tissue remodeling, and pro-hormone/enzyme cleavage. Notably, CPA3 is a characteristic feature of MCtc (MC type containing tryptase, chymase, and CPA3), which allows it to distinguish this type of MC from MCt [4,13,42].

Another vital substance stored in mast cell granules is histamine. It is an essential mediator, acting via four receptors (HR1-4). All of them have vastly different effects. HR-2 is a receptor antagonizing functions of HR1. It also reduces histamine release in basophils and MCs. However, studies on respective gene-deficient mice show that HR1- and HR2-deficient mice are unprotected from auto-antibody-induced arthritis. HR3 regulates the release of histamine and the negative feedback mechanism responsible for reducing central histaminergic activity. There is seemingly an HR-3-controlled mast-cell–neuron feedback loop. HR-4, a receptor very similar to HR-3, is involved in the recruitment and activation of inflammatory cells like mast cells, neutrophils, eosinophils, dendritic cells, and T cells. Thus, it is perceived as the immune system histamine receptor. Interestingly, HR-4 attracts mast cells toward histamine without causing their degranulation. In the K/B×N-antibody-induced arthritis model administration of the HR4 antagonist, clozapine protects mice from arthritic symptoms, underscoring its critical role as the primary histamine receptor in arthritis. On the other hand, inhibiting HR-3 has a slight initial effect on the mentioned disorder. However, it is important to note that the HR-3 antagonist, thioperamide, partially blocks HR-4, as it shares about 40% homology with HR3 [43,44].

### 3.6. Dependencies between MCs and Other Immune Cells in RA

In recent years, it has been proven that MCs can affect T lymphocytes in several ways. One of the well-known functionalities of MCs is the ability to present antigens to CD4+ T-cells. Mast-cell-derived cytokines can also induce T-cell activation. It has also been proven that the interaction between T helper cells and mast cells not only activates T-cells but can also change MC phenotype; moreover, regulatory T-cells can even inhibit MC activation [19].

In vitro studies suggest that activated MCs can also increase B cell activation, proliferation, and differentiation into IgM- and IgG-producing B cells, as well as enhance their antigen-presenting capacity. There is no certainty about the exact mechanism by which MCs induce B cells, but one of the confirmed means is cell contact and, specifically, CD40L–CD40 interaction [20,45]. Another proposed means of B cell activation by MC is Bruton’s tyrosine kinase (BTK), which acts as a proinflammatory in the synovium of RA patients. Activated BTK signaling can also increase the expression of proinflammatory cytokines, chemokines, and cell adhesion molecules [46,47].

In RA synovial tissue, human synovial MCs can be found close to CD141 monocyte/macrophages, CD31 T cells, and CD201 B cells. They were most commonly spotted near macrophages. MCs activated with IL-33 and IgG through the release of IL-10 and histamine were able to suppress monocyte activation by inhibiting both TNF production and up-regulation of the costimulatory molecule CD80 [25].

The impact of mast cells on inflammation, achieved through increased vascular permeability and the secretion of chemokines, which in turn leads to the infiltration of neutrophils and other immune cells, is well established. In rheumatoid arthritis, neutrophil chemoattraction to the synovial fluid is mainly mediated by IL-8, which is primarily secreted by mast cells in response to ACPA autoantibodies and TLR ligands. It is suspected that mast cells are helpful not only in enhancing lymphocytic infiltration but also in supporting the maintenance of specific lymphocyte subtypes by secreting growth factors like GM-CSF and G-CSF [19]. Interestingly, studies conducted on DBA/1J mice after immunization with bovine type II collagen suggest that the aforementioned neutrophil influx might vary with time across different joints [48].

### 3.7. Synovial Fibroblasts and Chondrocytes

In rheumatoid arthritis, chronic inflammation can trigger the activation of synovial fibroblasts. This activation, in turn, leads to increased invasiveness toward the cartilage tissue. Mast cells produce cytokines such as TNFα, IL-1, and IL-17, which can be engaged in synovial fibroblast activation. Furthermore, MCs, through the secretion of histamine and tryptase, regulate the apoptosis of these cells and thus may also affect their invasiveness. The interaction between MCs and synovial fibroblasts (SFs) can be bilateral through the secretion of stem cell factor and IL-33 by SFs, leading to MCs’ recruitment and activation [19,49].

Uncontrolled synovitis leads to the progressive destruction of joint structures, including cartilage and bone. Thus, chronic inflammation, activation of synovial fibroblasts, tissue remodeling, and angiogenesis are strictly associated processes in rheumatoid arthritis. In RA, angiogenesis occurs predominantly in the joint’s synovial lining because proliferating fibroblasts and other cells require more oxygen and nutrients. To our best knowledge, no data directly link mast cells and synovial angiogenesis. Still, mast cells are often positioned near blood vessels and secrete heparin, chymase, and tryptase—mediators that can contribute to the process [19].

Apart from synovial fibroblasts, chondrocytes are another kind of cell engaged in joint remodeling processes in RA. Both cells release MMPs, which degrade several extracellular matrix proteins, such as collagen and aggrecan. To become an active form, MMPs have to be cleaved by a proteinase. One of the enzymes known for such ability is MC-released tryptase. Another aspect of the remodeling process in RA is bone erosion. There is a possibility that mast cells accelerate bone turnover, likely through a direct effect of histamine on osteoclasts and indirectly through the production of tissue-destructive cytokines [19,32].

### 3.8. Dual Role of MCs in RA

The findings derived from animal studies provide compelling evidence of the substantial influence of MCs on arthritis. However, it is essential to acknowledge that certain aspects, such as the chronicity and heterogeneity observed in human RA, cannot be fully replicated in animal models. In vitro experiments also come with their limitations, as they involve indirect approaches. Many of the laboratory evidence supports the hypothesis of a proinflammatory role of MCs in RA. Still, depending on the type of environment and triggers, they can also exert immunomodulatory effects, suppressing innate and adaptive immune responses in various contexts, mainly via production of IL-10. For example, activated MCs induce TNF-α production, and IL-33 synergistically enhances its production—therefore, activation of MCs may exaggerate inflammation in RA. Nevertheless, IL-33- and immune-complex-triggered MC activation leads to the down-regulation of monocyte-mediated immune responses via IL-10 and histamine release, indicating that MCs might have anti-inflammatory functions [50,51]. The list of contradictions is extensive. For example, degranulated MCs are associated with RA severity, but some studies claim that mRNA levels of MC-specific genes are inversely associated with disease severity [52]. MCs also revealed a significant negative correlation with CLP1, an effective RA diagnostic marker. Moreover, in patients with early RA, there is a negative correlation between C-reactive protein and serum tryptase, a systemic marker of mast cell activation. Therefore, MCs might have an anti-inflammatory role in RA [53].

Such findings are consistent with Mishima et al.’s study results. This paper describes that synovial MCs from RA patients have substantially higher prostaglandin synthetase (PTGS)1 and PTGS2 expressions. MCs are assumed to be the only cells producing prostaglandin D2 (PGD2) in RA. PGD2 induces IL-8 production by human group 2 innate lymphoid cells, which may induce neutrophil recruitment into the synovium of RA patients. PGD2 spontaneously undergoes non-enzymatic dehydration to 15-deoxy-Δ12,14-prostaglandin J2 (15d-PGJ2). 15d-PGJ2 can inhibit NFκB signaling and cytokine release and act as an agonist of PPARγ. Therefore, by producing PGD2, MC might be both an anti-inflammatory and proinflammatory factor [54]. This situation is similar with regard to neurotransmitters, as neural pathways are thought to be directly involved in the pathogenesis of RA. Whereas neuromedin U is a neuropeptide with the proinflammatory activity that directly activates mast cells [55], the substance P (SP) function in the RA is more complex. SP is a peptide neurotransmitter responsible for pain signals and inflammation. Its proinflammatory effects are mediated by neurokinin-1 receptors (NK-1R). Not only nerve fibers but also MCs may be the source of tachykinin substance P in the joints of RA patients. SP activates synovial MCs to release histamine and proteases and to produce PGD2 but not IL-6 and TNF-α. The released SP is rapidly degraded by MC-derived chymase, which may downregulate the SP-mediated activation of synoviocytes in RA. Therefore, SP features both pro- and anti-inflammatory functions [56]. 

The last of the MC signaling pathways that are not detrimental in the context of RA is associated with dopamine, a catecholamine neurotransmitter. Dopamine receptors are present in various cells of the immune system, enabling dopamine to regulate essential processes such as proliferation, activation, adhesion, migration, differentiation, and apoptosis by binding to specific receptor subtypes. Dopamine receptors (DARs) are divided into two main types: D1-like DARs and D2-like DARs. Activation of D1-like DARs results in an increase in intracellular cAMP levels, while D2-like DARs suppress intracellular cAMP. Among the D2-like receptors, DAR-3 stands out with the highest affinity for dopamine among all DARs. Mast cells also express such receptors; hence, changing the level of D3R-positive MCs in the synovial fluid is not very unexpected. Indeed, D3R-positive MC level is correlated with disease severity DAS28 score in RA patients; the higher the score, the lower the level of D3R-positive MCs in the synovial fluid. Moreover, such MCs are positively correlated with the amount of antioxidants and negatively correlated with antioxidants. Wang et al. suggested that D3R inhibits mast cell inflammation by activation of the mTOR/AKT/AMPK-LC3-ubiquitin signaling axis, leading to TLR4 degradation. These findings indicate that D3R on MCs may play a protective role in RA patients. Nonetheless, it is still unknown if the lower expression of D3R on MCs is the cause or effect of ROS overproduction in RA patients [57,58].

All of these findings lead to the conclusion that more attention should be paid to the MC’s subtype when analyzing the impact of MC on diseases. Besides this, it can also be observed that MC activation by neurotransmitters can also give intriguing results.

## 4. Spondyloarthritis

Spondyloarthritis (SpA) is a group of the most prevalent chronic inflammatory arthritis, which significantly negatively impacts joints and tissues such as the skin, eye, and gut. The critical factor in this debilitating condition is IL-17A, which activates T cells and starts an inflammatory response [59]. There are contradictory findings regarding the origin of IL-17. Some of the studies prove its production directly by MCs, while studies conducted on mice failed to demonstrate that. Research conducted on human-derived cells report that MCs only capture exogenous IL-17 and release it via degranulation during inflammation. Interestingly, mastocytosis and spondyloarthritis show the same symptoms, such as gastrointestinal manifestations (bowel movements, diarrhea, epigastric pain and fullness, nausea, vomiting), joint pain, tendinitis, and osteoporosis [60,61,62].

Studies show significantly higher concentrations of MCs, IL-17A, CD 15+ neutrophils, and T-cells in synovial tissue and other SpA target tissues, such as the skin and gut, compared to healthy patients; in this article, Noordenbos et al. stated that mast cells are the main IL-17-expressing cell population in SpA, which has been confirmed by targeting and forcing the apoptosis of rheumatoid synovial mast cells with imatinib mesylate, which down-regulates the concentration of IL-17A and indirectly other proinflammatory cytokines, such as IL-6 and IL-8 [62].

Similar findings were reported in ankylosing spondylitis (AS), which is considered to be a subtype of SpA [62,63]. It is a chronic, genetic-related inflammatory disease primarily involving the mid-axis skeleton, leading to bone erosion, new bone formation, and ankylosis of the spine, all irreversible [64,65]. Research has also demonstrated that IL-23 can stimulate tryptase-positive mast cells to release IL-17, which, in turn, stimulates other immune cells. Interestingly, a high concentration of these mast cells was found in the fibrous tissue of aAS patients. IL-23-mediated tissue fibrosis has also been observed in individuals with ulcerative colitis and pulmonary fibrosis. Depending on the specific environment, IL-23 may exhibit either anti-inflammatory or proinflammatory effects. There is a suspicion that IL-23, by stimulating IL-22 secretion, could lead to the transformation of fibrous tissue into bone tissue, as it has the capacity to activate fibroblasts. In fact, IL-23 is secreted by the fibrous tissue of bones in patients with osteoarthritis, suggesting that this mechanism may apply to other disease entities as well [65].

## 5. Psoriatic Arthritis

Psoriatic arthritis is a chronic immunological-related disease in which chronic inflammation, and mast cell and keratinocyte activation cause a thickening and scaling of the skin [66]. One of the critical factors responsible for this pathological condition is IL-17A, which is released from mast cells via degranulation and the formation of extracellular traps in response to trauma or microbial infection [66,67]. IL-17 acting with IL-22 is responsible for inflammation, bone remodeling, and the release of the CXCL-family of cytokines and IL-8, which promotes neutrophil recruitment and migration into joints and the epidermis. This effect, in turn, contributes to the recruitment of CD8+ T cells (Tc17) and CCR6^+^ CD4+ T cells, which produces even more IL-17A, creating a self-propelled negative spiral [66,68,69].

Mast cells are stimulated and recruited by IL-33 recruit and stimulate MC, which are in turn responsible for releasing IL-17A, expressing and producing tryptase and trypsin-like serine protease. Tryptase plays an essential role in producing and releasing important proinflammatory cytokines such as IL-1β, IL-8, TNFα, and IL-6. It also stimulates the migration of leukocytes into joints by proteinase-activated receptor-2 (PAR-2), further enhancing inflammation, giving rise to the activation of immune cells. In addition to that, tryptase degrades the joint matrix by activating matrix metalloproteinases (MMPs) and latent collagenase, contributing to synovial hypertrophy [68,70].

In all skin and synovial fluid samples collected from patients diagnosed with psoriatic arthritis, MC and IL-17A concentrations were significantly increased compared to patients with rheumatoid arthritis [68,69,71]. A similar effect of excess mast cells in the synovial fluid caused by inflammation was found in patients diagnosed with gout.

## 6. Gout

Gout is an inflammatory and extremely painful arthritis with relapsing attacks [72,73]. It is characterized by monosodium urate (MSU) crystal deposition in the joints and periarticular tissues. The acute gout flare causes joints to become red, warm, swollen, and painful, which are all indicators of an acute inflammatory response. It is initiated by infiltration of the neutrophils followed by MSU crystal phagocytosis and subsequently by the release of proinflammatory mediators, such as IL-1β, into the synovial fluid [73,74]. This affection is very severe and debilitating. Often due to mechanical allodynia, patients cannot endure even being slightly touched at the affected joint, thus resulting in a worse quality of life [73].

Mast cells are activated by monosodium urate crystals and vanilloid receptor 1 (TRPV1)—an ion channel receptor expressed in MCs, which is induced by heat, protons, and lipid mediators [73,74]. These factors cause MCs to degranulate and secrete mediators such as histamine, tryptase, and many cytokines, including IL-1β, leading to nociception and inflammations in articular tissue [73,75]. It was established that tryptase and histamine were present in synovial fluid samples collected from gout patients with levels similar to specimens taken from subjects with active rheumatoid arthritis; however, the concentration of IL-1β was significantly higher in gout patients, confirming the significant role of this cytokine and thus a vital function of mast cells in gouty inflammation [74]. Moreover, it has been reported that many mast cells are present in all gout tophi [76]. It was proven that ketotifen notably inhibited mast cell activation and decreased histamine concentrations and other proinflammatory mediators such as nitric oxide, interleukin-1β, and interleukin-6 in samples collected for rats with gout. This proves that ketotifen may attenuate MSU-induced acute inflammation [72]. Furthermore, because of the significant role of TRPV1 activation, it may become a potential target for developing new therapies to treat acute gout attacks [73].

## 7. Injuries

Injuries are one of the most common musculoskeletal problems. Major joint injuries and post-traumatic contractures are both functionally debilitating complications. Surgical procedures to improve elbow motion are required in 12–15% of elbow injuries. In most cases, surgical procedures are used to enhance joint motion. This incorporates removing the joint capsule; nonetheless, the normal range of motion is never fully restored [77].

A significant increase in the number of mast cells, along with neuropeptides and myofibroblasts, has been observed in joint capsule samples taken from patients with post-traumatic contractures, compared to regular joint capsules. Notably, the use of ketotifen, a mast cell stabilizer, has been found to prevent the release of bioactive factors, leading to a decrease in the number of myofibroblasts, mast cells, and fibrosis. These findings suggest that an abnormal myofibroblast–mast cell–neuropeptide axis may play a role in the pathological changes observed in the joint capsule during post-traumatic contractures. Mast cells are critical factors in myofibroblast activation, leading to collagen matrix synthesis, attachment, and contraction [77].

A similar effect has been reported in intervertebral disk injury convalescence. In this process, MCs, peripheral cells, interlamellar cells, progenitor cells, macrophages, and T lymphocytes are involved in healing, including inflammation and cell proliferation, leading to matrix remodeling [78].

## 8. Intervertebral Disc Degeneration

Intervertebral disc degeneration (IDD) is a typical degenerative disease causing low back pain (LBP), a medical disorder associated with disability, high socioeconomic expenditure, and unsatisfactory long-lasting therapeutic effects. Although the etiology of IDD is considered to be multifactorial, ranging from genetic predisposition and ageing to lifestyle, this condition is characterized by extracellular matrix degradation and excessive cell apoptosis, attributed to inflammation with the recruitment of immune cells such as CD4+, CD8+ T-cells, T-regs, neutrophils, macrophages, and mast cells and inflammatory cytokines including IL-1*β* and TNF-*α*. TNF-*α* has a significant effect within the disc and promotes the death of the nucleus pulposus (NP)—the largest avascular organ located at the center of the intervertebral disc (IVD), thus making it an immune-privileged organ. Disruption of the NP–blood barrier due to annulus fibrosus rupture leads to exposure of the NP to the host and triggers immune cell infiltration and response [79,80]. An increase in ROS in the intervertebral disk can cause oxidative damage to disk cells, activation of NLRP3 inflammatory vesicles, and increased IL-1 release, intensifying the inflammatory response. Studies have shown that concentrations of mast cells were significantly higher, while the proportion of CD4+ cells, NK-activated cells, and macrophage M0 was notably lower in IDD patients compared to the control group. It was found that MCs have a significant role in the repair of the injured annulus fibrosus and subsequent disc degeneration, thus explaining why these cells are significantly increased in IDD [79,80]. The related role of MCs in tissue repair was also found in muscle damage.

## 9. Muscles

Eccentric muscle contractions often result in ultrastructural alterations in muscle tissue, often discovered in unaccustomed and intense exercise. The time required to recover from the aforementioned muscle damage hinges on several factors, such as initial muscle damage, joint angle or muscle length, and muscle groups used during activity. It is well known that inflammation is essential for muscle recovery; thus, tightly regulated inflammatory responses are integral to muscle repair and regeneration [81].

Various immunological cells, mast cells, fibro-adipogenic progenitors, and pericytes are involved in muscle tissue regeneration. The recovery period is initiated by neutrophils and macrophages, which are tasked with clearing cellular debris and cytokine secretion to increase inflammation. Mast cells also infiltrate muscle tissue, releasing histamine, chemoattractants, and tryptase, which increases myoblast proliferation and decreases myoblast differentiation [81,82].

Treg cells, similar to mast cells, stop myoblast differentiation and fibrosis. After that, proinflammatory factors are replaced with CD8+ and T-regulatory lymphocytes, which facilitate muscle regeneration by stimulating the secretion of CCL2 and recruiting M1-like macrophages into the muscle. Eosinophils secrete the anti-inflammatory cytokine IL-4, stimulating fibro-adipogenic progenitors to initiate myoblast differentiation and necrosis. Lastly, type 2 pericytes secrete various growth factors that enhance myoblast differentiation while also stimulating satellite cell quiescence. Other stromal cells, including fibro-adipogenic progenitors and pericytes, are activated and support myoblast differentiation. If this process is efficient, muscle fibers’ ultrastructure is restored in around seven days [81].

This process, however, is not always beneficial. Muscle-resident immune cells, including MCs, are involved in cancer-associated cachexia. In a study involving mice with moderate and severe cachexia, there was a significant difference in the tryptase alpha/beta 1 (TPSAB1) and CD68 genes from the innate immune system compared to healthy mice, serving as a control group. TPSAB1 is associated with MCs, while CD68 is a macrophage marker. TPSAB1 expression was crucially increased in cathartic mice, and TPSB2 expression in early cachectic mice was notably higher compared to healthy mice. This study suggests that mast cells and macrophages may be crucial in cancer-associated cachexia [83].

## 10. Tendinopathy

Tendinopathy is a pathological condition of tendon tissue. It is connected with remodeling in major body tendons, characterized by abnormalities (tendon thickening) in the molecular structure of the cell matrix, and accompanied by excessive nociceptive signaling [84,85]. It is also often associated with high healthcare costs because there are no symptoms in the initial development of tendon pathology [86].

Tendinopathies commonly cause pain and disability in athletes [87]. In most studies relating to tendinopathies, including non-ruptured chronic tendinopathic Achilles tendon samples, an increased number of mast cells have been found [88]. Upon activation, those cells release histamine, tryptase, and cytokines such as TGF-beta and VEGF [84]. Through this effect, mast cells contribute to tissue repair in injured soft tissues. The whole process is initiated by binding substance P (SP) to its receptors, NK1R, on mast cells, causing the release of the formerly mentioned substances contained in mast cell granules and stimulating de novo leukotriene and prostaglandin production, in turn leading to mediating pain, oedema, and fibrosis—common findings in chronically painful tendons [86,89].

Apart from secreting histamine, tryptase, and cytokines, mast cells, along with fibroblasts, neurons, and endothelial cells, express Protease-Activated Receptors. In vivo, the best-known activators of PARs are tryptase, thrombin, and above all, trypsin. Mast cells, as was mentioned earlier, contain high concentrations of trypsin, thus acting as a primal activator of PARs. The best-known receptor involved in tendinosis is PAR-2. It is responsible for fibroblast proliferation, angiogenesis, hyperalgesia, changes in collagen expression, and the local excretion of SP. Since PAR-2 activation is initiated by tryptase, and there is a significant increase in mast cell numbers in injured tendon tissue, it is logical to assume that this receptor, and therefore MCs, is one of the vital factors in tendon pathology [84].

## 11. Heterotopic Ossification

Heterotopic ossification (HO) is a disabling condition associated with neurologic injury, inflammation, overactive bone morphogenetic protein (BMP) signaling, and poor quality of life. A type of ultra-rare progressive genetic, very severe, and debilitating type of heterotopic ossification is called fibrodysplasia ossificans progressiva (FOP). It progresses in stages—firstly, there is a soft tissue response with early inflammatory connective tissue destruction, followed by fibroproliferative cell expansion. It is caused by heterozygous mutations in Activin receptor A type I (*ACVR1*). An overwhelming 95% of patients possess the R206H mutation in *ACVR1*, which is responsible for conferring ligand-independent and ligand-responsive gain-of-function BMP signaling [90,91]. Both HO and FOP are commonly marked by “flare-ups” described as swelling, stiffness followed by pain, and warmth felt in soft connective tissues after any type of tissue injury [90,92].

It was found that the number of immune cells participating in the normal tissue injury response is significantly increased in *Acvr1^cR206H/+^* lesions and remains at high levels instead of returning to preinjury levels as occurs during wound repair. High cell level is accompanied by increased proinflammatory factors, which alter proinflammatory cytokines released by mast cells. This fact suggests that mast cells play a crucial role in promoting HO and FOP. Studies determined that the depletion of macrophages or/and MCs in *Acvr1^cR206H/+^* mice significantly reduced HO effects. Both MCs and macrophages secrete IL-6 and TNF-α, and the inflammatory process is diminished by depleting one of both cell populations. Reducing the number of mast cells and macrophages also alters inflammatory signaling with other immune cells, attributed to the lower proinflammatory cytokine expression in the remaining immune cells and contributing to the reduced HO seen in mast-cell-depleted and/or macrophage-depleted *Acvr1*^cR206H/+^ mice models. Furthermore, reducing proinflammatory cytokines enables the expression of anti-inflammatory cytokines, such as IL-4, IL-10, IL-13, and TGF-β, thus promoting tissue regeneration. Another factor in HO and FOP is the BMP signaling pathway, which has multiple distinct roles during skeletal muscle injury and repair. BMP ligands are responsible for inducing the production of inflammatory cytokines and edema, both of which occur prior to the initiation of HO in FOP patients. Moreover, BMP signaling activates macrophages and T cells, leading to the upregulation of P57 expression, which in turn stimulates mast cell degranulation, resulting in the expression of TNFα and IL-6 [90,92,93]. These findings strongly indicate that mast cells (MCs) play a significant role in the HO and FOP.

## 12. Ehlers–Danlos Syndrome

Ehlers–Danlos syndrome (EDS) is a heterogeneous group of heritable connective tissue disorders that share common features, such as hyperextensibility of the skin, joint hypermobility, and tissue fragility. The first recorded observations of a disease with symptoms consistent with EDS were made in 400 BC by Hippocrates. At the beginning of the 21st century, the disease was given an official name based on the names of the leading researchers who described it [94]. The current classification, established in 2017, recognizes 13 subtypes of EDS, with the hypermobility type (hEDS) being the most prevalent. The genetic basis for most types of EDS is known, except for the hEDS. Most of the identified genes are associated with collagen structure and assembly or cross-linking of the procollagen fibrils. The frequency of all types of Ehlers–Danlos syndrome has been estimated to be at a minimum of 1 in 5000 individuals. Among EDS patients, the hypermobility type is believed to account for up to 80–90% of all EDS patients [95,96]. In this subtype, patients experience hypermobile and unstable joints, along with other signs of weakened connective tissues. Additionally, patients may encounter gastrointestinal symptoms, hypotension, fatigue, headaches, memory and concentration problems, among other issues, in addition to musculoskeletal complaints [95]. As a result, hEDS is currently considered a heterogeneous multisystem disease with many symptoms difficult to attribute only to improper connective tissue development [94].

Hypermobile EDS and Hypermobility Spectrum Disorder (HSD) exhibit highly similar symptoms, leading to the suspicion that they may share a common pathogenesis or even be variants of the same disorder. Moreover, individuals with EDS often experience various comorbidities. Among these, a potential association between EDS and Mast Cell Activation Disorders (MCADs) has been observed, particularly in patients with hEDS. MCAD comprises a group of immunological disorders characterized by an increased number of MCs, their increased activity of MCs, or both [97]. Currently, researchers are working to gain a better understanding of the pathogenesis and classification of this group of disorders, as well as to establish accurate diagnostic criteria. The MCAD subtype most often addressed in the context of hEDS is Mast Cell Activation Syndrome (MCAS).

MCAS is characterized by increased MC activation due to abnormal sensitivity but without MC proliferation. Its symptoms are similar but not consistent with allergy or systemic mastocytosis, and because it affects many organs, it is very heterogeneous and not specific. Therefore, symptoms may include flushing, pruritus, urticaria, angioedema, nasal congestion, nasal pruritus, wheezing, throat swelling, headache, diarrhea, abdominal cramping, unexplained arrhythmias, and hypotensive episodes [95,98]. Some studies reported that up to 25% of patients with hEDS also have MCAS [96]; however, such associations raise several concerns [95]. In order to understand the attributed links between MC and EDS, it is worth familiarizing oneself with the information that has made researchers lean towards such hypotheses.

First, it is essential to note the similarities that characterize both hEDS and MCAD. Hypermobile EDS is no longer perceived as a musculoskeletal disease affecting only connective tissue. It is a multisystem, heterogeneous disease, which—just like MCADs—produces symptoms also related to the digestive system, skin, pain sensation, chronic fatigue, as well as autonomic dysfunction [99,100,101]. Some of these symptoms are difficult to exclusively attribute to abnormal connective tissue, which justifies suspicions of additional factors contributing to the nature of hEDS. Regarding the disproportion in patients’ sex, the vast majority of patients in both conditions are female. Also, hEDS and MCAS are associated with a recently recognized condition, hereditary alpha-tryptasemia, characterized by overexpression of tryptase [99]. The MCAD and hEDS connection may be rooted in the release of MC mediators. Tryptase and histamine are of central interest in this case, as they are involved in the fibroblast-to-myofibroblast transition, proliferation, and collagen production [101].

In hEDS, immune system regulation might be altered, as MCs are responsible for detecting and responding to tissue injury. When connective tissue is injured, MCs are activated by Damage-Associated Molecular Patterns (DAMPs), also known as alarmins. The most important ones are thymical stromal lymphoprotein (TSLP) and IL-33. MCs release corresponding mediators based on a combination of detected factors [94]. Because of all of the information mentioned above, a concept emerged that in hEDS and HSD, mediators from at least MCs, whether primary to one of the MCADs, or other disorders, lead to the production of abnormal connective tissue [99]. However, even if this hypothesis is true, it would not clarify if MCs initiate this disorder or if they only intensify it.

MC-related disorders might be rooted in more than only hEDS. A survey conducted on 3276 participants with POTS concluded that 25% of them have EDS and 9% have MCAS, which may suggest some connection between those entities. In another questionnaire, 66% of the patients diagnosed with both POTS and EDS reported symptoms suggestive of MCAS [102].

Indeed, the diagnosis of both MCADs and hEDS can be challenging. For instance, laboratory tests for MCAD should focus on identifying the levels of at least two mediators at two different time points, preferably when the patient is experiencing significant symptoms. From over 100 MC mediators, only a few can be tested in commercial laboratories—and even within that group, the sensitivity of the tests cannot be high enough [97]. In the case of hEDS, most of the problems are caused by the diagnostic criteria, affecting the homogeneity of diagnosed patients and thus the possibility of finding common underlying factors or relationships between different disease entities. More strict criteria were proposed to deal with this dilemma; another proposal was to distinguish a spectrum of hypermobility disorders, with hEDS at the more severe end of such spectrum [95].

That is why some scientists do not support the MCAS-hEDS association, as there is too a broad spectrum of symptoms attributed to the hEDS, and there is no sufficient number of objective tests for MCADs and limited knowledge about its pathophysiology [95,96]. Also, many published articles were studied in too small a groups, and some questionnaires were not standardized [100]. Other researchers emphasize that MC mediators, like tryptase, histamine, prostaglandin D2, and leukotriene D4, increase fibroblast proliferation and collagen production. Even though so many scientists state that there is no sufficient evidence for the association between MCADs and hEDS right now, such a correlation cannot be ruled out. It only means that such studies should be conducted on larger groups of patients diagnosed with more strict criteria to prove the mentioned findings [95].

## 13. Conclusions

Recently, the spotlight has increasingly turned to the role of mast cells in the pathogenesis of diseases. Researchers now recognize MCs as crucial factors that can significantly impact the development of various disorders, ranging from musculoskeletal issues to immune-related conditions. This article presents convincing evidence supporting the importance of this field-related research, as it helps gain a better understanding of the nature of many pathologies. However, as evidenced in the paper, it also emphasizes the need for further extensive research and a re-evaluation of some of the previously published reports to unlock the full potential of this field.

While reading this review, it is important to acknowledge certain limitations. The selection of literature was conducted by two researchers, striving to maintain objectivity and adherence to guidelines. Nevertheless, complete unbiasedness may not be achievable. Furthermore, the inaccessibility of three articles during the literature selection process may have impacted the final description of certain aspects discussed in this review.

In conclusion, exploring the role of MCs in the pathogenesis of musculoskeletal diseases is a promising avenue that warrants further investigation. By overcoming the acknowledged limitations and conducting robust research, invaluable insights can be uncovered, which may revolutionize our understanding and treatment of various disorders.

## Data Availability

All necessary data are included in the paper.
